# Preterm subtypes by immigrants’ length of residence in Norway: a population-based study

**DOI:** 10.1186/1471-2393-14-239

**Published:** 2014-07-21

**Authors:** Ingvil K Sørbye, Anne K Daltveit, Johanne Sundby, Siri Vangen

**Affiliations:** 1 Norwegian Resource Centre for Women’s Health, Women and Children’s Division, Oslo University Hospital, P.O. Box 4950 Nydalen, Oslo 0424, Norway; 2 Norwegian Institute of Public Health, Oslo, Norway; 3 Department of Global Public Health and Primary Care, University of Bergen, Bergen, Norway; 4 Institute of Health and Society, University of Oslo, Oslo, Norway

**Keywords:** Preterm delivery, Immigrants, Length of residence, Country of birth, Spontaneous preterm delivery, Non-spontaneous preterm delivery, Iatrogenic preterm delivery

## Abstract

**Background:**

The reduction of the preterm delivery (PTD) rate is a maternal and child health target. Elevated rates have been found among several immigrant groups, but few studies have distinguished between PTD according to the mode of birth start. In addition, migrants’ birth outcomes have further been shown to be affected by the time in residence; however, the association to PTD subtypes has not previously been assessed. In this study we examined if the risk of spontaneous and non-spontaneous, or iatrogenic, PTD among immigrants in Norway varied according to the length of residence and the country of birth, and compared with the risks among the majority population.

**Methods:**

We linked population-based birth and immigration data for 40 709 singletons born to immigrant women from Iraq, Pakistan, the Philippines, Somalia, Sri Lanka and Vietnam and 868 832 singletons born to non-immigrant women from 1990–2009. Associations between the length of residence and subtypes of PTD were estimated as relative risks (RRs) with 95% confidence intervals (CIs) from multivariable models.

**Results:**

In total, 48 191 preterm births occurred. Both spontaneous and non-spontaneous PTD rates were higher among immigrants (4.8% and 2.0%) than among non-immigrants (3.6% and 1.6%). Only non-spontaneous PTD was associated with longer lengths of residence (p trend <0.001). Recent immigrants (<5 years of residence) and non-immigrants had a similar risk of non-spontaneous PTD, whereas immigrants with lengths of residence of 5–9 years, 10–14 years and ≥15 years had adjusted RRs of 1.18 [95% CI 1.03,1.35], 1.43 [95% CI 1.20,1.71] and 1.66 [95% CI 1.41,1.96]. The association was reduced after further adjustments for maternal and infant morbidity. Conversely, the risk of spontaneous PTD among immigrants was not mitigated by length of residence, but varied with country of birth according to the duration of pregnancy in term births.

**Conclusions:**

Non-spontaneous PTD increased with the length of residence whereas spontaneous PTD remained elevated regardless of the length of residence. Policies to improve birth outcomes in ethnically mixed populations should address the modifiable causes of PTD rather than aiming to reduce absolute PTD rates.

## Background

Preterm delivery (PTD) is strongly associated with perinatal mortality and morbidity [1]. As an indicator of adverse pregnancy outcome, a reduction of the PTD rate, in general, is a maternal and child health target. However, several subtypes of PTD have been identified, such as spontaneous PTD, resulting from spontaneous start of labour, and non-spontaneous, or iatrogenic, PTD, resulting from a medical intervention [2,3]. Although the two subtypes share certain determinants, a distinction has been recommended to clarify the mechanisms involved and to identify high-risk subgroups [4].

Preterm rates among migrants to high-income countries vary by the maternal country of origin [5-7]. In comparison to majority populations, migrants born in sub-Saharan Africa and Asia have higher PTD rates whereas other groups experience lower rates [8-12]. Ethnic variation has also been found in the duration of pregnancy *at term*[13,14]. However, the nature of this difference – whether physiological or pathological – remains contested [15,16]. Differences in the duration of pregnancy could potentially influence PTD rates, especially among groups with short gestational lengths. This would be more likely to influence spontaneous PTD rates. Few studies of ethnic disparities in the risk of PTD have differentiated between PTD subtypes and different methods for the determination of gestational length have been used [8,17].

**Figure 1:**
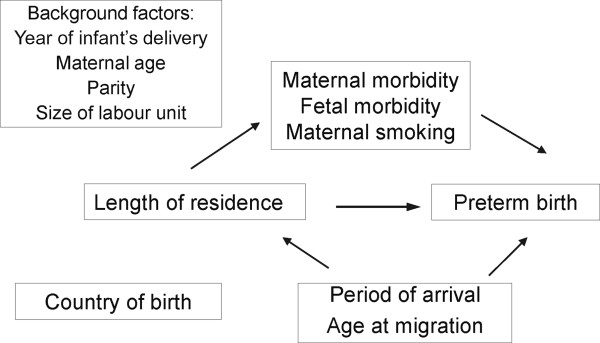
Study diagram.

Preterm rates in migrants could be affected by pre-migration exposures as well as by exposures after migration. Although a ‘healthy migrant’ effect in terms of infant outcomes has been shown among certain migrant populations, such effects could be lost with increasing exposure to the host country [18]. A proxy measure for the degree of exposure to the receiving country is the length of residence in the receiving country [19]. Longer length of residence has been associated to both risk factors and protective factors for PTD [20-23]. In pregnancy, length of residence has been associated to adverse maternal behaviour such as smoking [18,24]. Studies across generations of migrant origin to the US have shown that PTD or low birth weight rates rates among immigrant populations tend to converge towards the pattern observed in the native-born population [6]. A study from Canada found that the risk of PTD increased with longer length of residence and exceeded the risk among non-immigrants after 10 years of residence [25]. One Danish study found a U-shaped association between PTD and the length of residence [11]. Few studies of migrants to Europe have included length of residence in the study of PTD, and none of these studies have differentiated between PTD subtypes.Facing a steep increase in births to immigrant women in Europe, this study aimed to determine if the risk of spontaneous and non-spontaneous preterm delivery in Norway vary according to the length of residence and the country of birth. We hypothesised that with longer length of residence in Norway, migrants’ risk of both spontaneous and non-spontaneous PTD subtypes would increase and potentially surpass the risks in the receiving population. Furthermore, we speculated that the effect of length of residence on the risk of PTD subtypes could be mediated by changes in maternal and fetal morbidity and might be modified by demographic characteristics, enabling identification of subgroups at sustained elevated risk (Figure 1).

## Methods

### Data sources

We linked birth and immigration data for all births occurring during a 20 year period among the six largest immigrant groups and used births among non-immigrants as the reference. Norway was a suitable setting for this study because nearly all births in this country occur within the public health sector. We matched birth records dated between January 1^st^, 1990, and December 31^st^, 2009, from the Medical Birth Registry of Norway (MBRN) with files from the National Population Register. The birth registry includes demographic data, information on maternal health before and during pregnancy, gestational length at birth and birth outcomes. The National Population Register contain information on the mother’s country of birth and country of origin, her first date of immigration to Norway and her education level. Unique personal identification numbers enabled linking of the two sets of registry data. The MBRN granted access to the birth data and the Regional Ethics Committee of Norway East approved the linkage to the Population Registry.

### Study population

We extracted data from records of singleton births among non-immigrant women and among immigrant women from six countries outside Scandinavia, which enabled an analysis by country of birth [5]. We excluded stillbirths that occurred at <28 completed gestational weeks (0.4%) and births that occurred before registered immigration (1.5%). We further excluded cases that were missing information on gestational length (3.8%) and cases with improbable birthweights based on gestational length and sex (z-scores >4 standard deviations (SDs) away from the majority standard; 0.5%) [26]. The final sample included 40 709 births among immigrants and 868 832 births among non-immigrants. The six minority groups comprised mothers from Pakistan (n = 10 096), Somalia (n = 8 094), Vietnam (n = 6 336), Iraq (n = 5 879), Sri Lanka (n = 5235) and the Philippines (n = 5 069).

### Outcome measures

PTD was defined as delivery between completed gestational week 22 + 0 days and week 36 + 6 days. We classified PTD by mode of onset into ‘spontaneous PTD’ or ‘non-spontaneous PTD.’ Non-spontaneous PTD included births that required medically induced labor or a cesarean section before labor. Spontaneous PTD included births that began spontaneously, including preterm labor or prelabor rupture of membranes. Between 1990 and 1998, gestational length was determined based on the last menstrual period (LMP) reported at the first antenatal visit. From 1999 onward, findings from an early second-trimester routine ultrasound were primarily used (97.5%) to determine gestational length, and the LMP was used when ultrasound data were unavailable [27].

### Main exposures

The main exposures were country of birth and length of residence. Country of birth was the country where the woman’s mother resided at the time of delivery. Length of residence was calculated as the interval between the year of first immigration to Norway and the year of delivery. In the final analyses the length of residence was categorized as 0–4, 5–9, 10–14 or ≥15 years. The first category (0–4 years) was further subdivided into <2 years and ≥2 years; however, estimates were the same for the two subcategories, so the subcategories were subsequently collapsed.

### Covariates

Based on the available literature, we selected co-factors associated with the length of residence and PTD [2,28]. We extracted data on the following variables:

• year of infant’s delivery

• period of births, categorized as ‘1990-1998’ and ‘1999-2009’

• maternal age at the time of delivery, in years

• parity, defined as the number of previous deliveries

• the size of the labor unit, as proxy for urban/rural residence

• maternal smoking status before/ during pregnancy

• age at migration, categorized as quartiles

• period of arrival, categorized as ‘<1993’ or ‘≥1993’

• gestational or pregestational diabetes

• hypertensive disorders, including pregnancy-induced hypertension, preeclampsia and eclampsia

• placenta previa

• placental abruption

• major birth defects

• small- for-gestational age (SGA) and large-for-gestational age (LGA) infants (5^th^ and 95^th^ percentiles, respectively, of the majority standard) as proxies for abnormal fetal growth [26].

### Statistical methods

The means of the covariates were compared using the t-test/analysis of variance (ANOVA). Categorical covariates were compared using the χ^2^ test and the χ^2^ test for trends. To directly assess the relative risks (RRs) of the outcomes with 95% confidence intervals (CIs) we used univariable and multivariable Poisson regression in generalized linear models to estimate the associations between the length of residence and subtypes of PTD. Robust variance estimator (Huber-White) was applied to the models to adjust for data dependencies in mothers who gave birth more than once. See Tables 1 and 2 for categorizations of the covariates.

**Table 1:** Demographic covariates in non-immigrants and in immigrants, by the length of residence, Norway 1990-2009

**Non-immigrants**		**Immigrants**	**Immigrants by the length of residence (years)**				
**Births (%)**	**868 832 (95.5%)**	**40 709 (4.5%)**	**20 074 (49.3%)**	**11 077 (27.2%)**	**4926 (12.1%)**	**4632 (11.4%)**	
			**0-4**	**5-9**	**10-14**	**≥15**	
	**%**	**%**	**%**	**%**	**%**	**%**	**P**^**a**^
Country of origin							<0.001
Iraq		14.4	20.0	12.9	6.9	2.1	
Pakistan		24.8	19.0	22.1	31.2	49.5	
Philippines		12.5	14.6	11.6	10.4	7.2	
Somalia		19.9	22.3	24.5	14.4	4.2	
Sri Lanka		12.9	12.7	15.7	13.7	5.9	
Vietnam		15.6	11.4	13.2	23.3	31.0	
Period of birth							<0.001
1990-1998	46.1	29.5*	31.0	31.8	30.0	17.4	
1999-2009	53.9	70.5	69.0	68.2	70.0	82.6	
Age at migration							<0.001
<20 years		24.5	7.7	20.9	46.6	86.5	
≥20 years		75.1	92.3	79.1	53.4	13.5	
Period of arrival							<0.001
Before 1993		37.4	16.6	36.0	69.4	97.1	
1993 onwards		62.6	83.4	64.0	30.6	2.9	
Maternal age (y)							<0.001
<20	2.7	2.2*	2.8	1.6	1.9	1.0	
20-24	17.3	21.1	28.9	12.9	12.9	15.9	
25-29	35.2	34.5	37.4	35.0	23.9	31.7	
30-34	30.6	27.4	21.8	33.7	34.8	28.4	
35-39	12.2	12.1	7.6	14.2	21.6	17.0	
≥40	1.9	2.7	1.5	2.5	5.0	6.0	
Parity							<0.001
0	41.2	34.1*	46.6	18.7	21.9	29.9	
1	36.0	29.4	30.0	30.7	24.1	28.8	
≥2	22.8	36.5	23.3	50.6	54.0	41.3	
Education level							<0.001
<12 years	56.3	56.1*	44.5	62.8	74.5	70.9	
≥12 years	43.6	12.0	8.7	12.1	14.9	23.2	
missing	0.1	31.9	46.8	25.1	10.6	5.9	
Size of labor unit							<0.001
<1500	37.6	15.6*	21.4	12.7	8.6	5.0	
1500-2999	28.1	30.8	31.2	30.2	32.0	29.5	
≥3000	34.3	53.6	47.4	57.1	59.4	65.5	

*Different from non-immigrants (χ^2^ test with P < 0.05). ^a^Associated to the length of residence (χ^2^ test with P < 0.05).

**Table 2:** Maternal and fetal health-related covariates in non-immigrants and in immigrants, by the length of residence, Norway 1990-2009

**Non-immigrants**		**Immigrants**	**Immigrants by the length of residence (years)**				
**Births (%)**	**868 832 (95.5%)**	**40 709 (4.5%)**	**20 074 (49.3%)**	**11 077 (27.2%)**	**4926 (12.1%)**	**4632 (11.4%)**	
			**0-4**	**5-9**	**10-14**	**≥15**	
	**%**	**%**	**%**	**%**	**%**	**%**	**P**^**a**^
Term gestational days, mean (SD)^b^	282 (9.0)	280 (9.4)	280 (9.4)	280 (9.3)	279 (9.1)	278 (9.0)	<0.001
Gestational diabetes	0.7	2.6*	1.7	3.1	3.3	4.3	<0.001
Pregest. diabetes	0.5	1.1*	0.6	1.2	1.9	2.2	<0.001
Hypertensive disorders	5.2	3.6*	3.3	3.5	4.5	4.2	<0.001
Abruptio placentae	0.5	0.5	0.5	0.4	0.5	0.5	0.829
Placenta previa	0.2	0.3*	0.3	0.4	0.3	0.5	0.235
SGA^c^	4.2	9.2*	9.7	8.1	8.0	10.3	<0.001
LGA^d^	6.3	3.0*	2.5	3.5	4.2	2.8	<0.001
Major birth defects	2.4	2.4	2.4	2.5	2.3	2.6	0.714
Smoking^e^							<0.001
Yes	18.9	1.6*	1.2	1.2	2.4	3.1	
No	61.6	67.0	68.5	66.2	65.9	63.9	
Not disclosed	19.5	31.4	30.3	32.6	31.7	33.0	

*Different from non-immigrants (χ^2^ test with P < 0.05). ^a^Associated to the length of residence, (χ^2^ test with P < 0.05). ^b^Term gestational days with standard deviation was calculated for term infants with spontaneous labor onset. ^c^SGA, small-for-gestational age infant, 5th percentile. ^d^LGA, large-for-gestational age infant, 95th percentile. ^e^Smoking was available from 1999 onwards.

First, we compared *non-immigrants with immigrants* with various lengths of residence. In the first model, we adjusted for demographic covariates which included the year of delivery, maternal age, parity and the size of the labor unit. In the second model, we adjusted for maternal and fetal health-related covariates which included gestational and pregestational diabetes, hypertensive disorder, smoking (‘yes’, ‘no’ and ‘undisclosed’), placenta disorders, SGA, LGA and major birth defects. Second, we applied the same models to *immigrants only*, where we also included the country of birth in the model. We used recent immigrants (0–4 years) as the reference. For these analyses among immigrants only, we needed a country reference group and chose Iraqi women, as this group had similar PTD rates as Norwegian women. Due to the relationships between age at the time of immigration, the year of arrival and the covariates already in the model, these variables were added separately to the model as categorical covariates; however, the pattern of the results remained unchanged. Interaction terms between the length of residence and age at immigration and between the length of residence and the country of birth were not significant. Models were checked for goodness of fit. A two-sided p-value of 0.05 was considered statistically significant. The analyses were conducted using SPSS version 20 (IBM Corp., Armonk, NY, USA).

### Sensitivity analyses

An application of the models to live births only did not change the pattern of results; this was also the case when we excluded major birth defects and when we restricted the sample to women living in the capital area. To reduce bias arising from the change in the method of determining gestational length, we limited the analyses to 1999–2009, which did not change the association pattern either. Moreover, in the analyses limited to immigrants, we stratified by early (gestational weeks 22–31) and late (gestational weeks 32–36) PTD to assess whether the relationship with the length of residence remained consistent.

## Results

### Demographic and health related characteristics

Immigrants differed from non-immigrants with respect to demographic covariates and these characteristics were also associated with the length of residence (Table 1). The number of births in each minority group varied with the length of residence, reflecting changes in migration patterns to Norway over the 20-year period. The six minority groups analyzed comprised 2.6% of births in Norway in 1990 and 7.4% of births in 2009. Women from Pakistan and Vietnam had the highest proportion of the longest residencies (≥15 years) before delivery, while women from Somalia and Iraq had the highest proportion of the shortest residencies (0–4 years). Maternal and fetal health-related covariates associated with immigrants’ length of residence were gestational diabetes, pregestational diabetes, hypertensive disorder and an SGA or LGA infant (Table 2). There were significant differences in the gestational length *at term* between country groups (Table 3). Five of the six minority groups had a shorter mean gestational length in term spontaneous births compared with non-immigrants. Vietnamese women had the shortest gestational length, which was 6 days shorter than the longest gestational length, which was observed among Somali women. Gestational and pregestational diabetes and giving birth to an SGA infant were more common among most immigrant groups, whereas hypertensive disorders and giving birth to an LGA infant occurred less often in immigrants compared with non-immigrants (Tables 2 and 3). In univariate analyses, the risk factors for PTD were mainly similar for immigrants and non-immigrants, except for the level of education, where a high education level was protective among non-immigrants, whilst there was no association between education level and PTD among immigrants (data not shown).

**Table 3:** Demographic and obstetric covariates by maternal country of birth, Norway 1990–2009

	**Non-immigrants**	**Immigrants**					
	**n = 868 832**	**Pakistan n = 10 096**	**Somalia n = 8094**	**Vietnam n = 6336**	**Iraq n = 5879**	**Sri Lanka n = 5235**	**Philippines n = 5069**
	**%**	**%**	**%**	**%**	**%**	**%**	**%**
Period of birth							
1990-1998	46.1	40.4*	16.8*	38.8*	8.7*	37.7*	32.3*
1999-2009	53.9	59.6	83.2	61.2	91.3	62.3	67.7
Age at migration							
<20 years		38.5	17.0	40.4	16.3	15.4	11.1
≥20 years		61.5	83.0	59.6	83.7	84.6	88.9
Period of arrival							
Before 1993		56.3	17.1	62.5	6.9	37.6	36.4
1993 onwards		43.7	82.9	37.5	93.1	62.4	63.6
Maternal age (y)							
<20	2.7	2.1*	3.1*	1.8*	3.6*	1.0*	0.9*
20-24	17.3	27.8	20.6	20.6	22.7	15.5	13.3
25-29	35.2	35.4	33.6	35.9	32.5	37.1	31.8
30-34	30.6	23.2	26.5	27.1	26.9	32.0	33.0
35-39	12.2	9.2	12.8	11.8	11.8	12.7	17.2
≥40	1.9	2.2	3.5	2.8	2.6	1.8	3.7
Parity							
0	41.2	28.8*	23.4*	39.8*	35.6*	41.0	45.9*
1	36.0	26.0	21.8	33.3	29.2	37.1	35.5
≥2	22.8	45.2	54.9	26.9	35.1	21.9	18.6
Education level							
<12 years	56.3	58.5	60.7	67.0	42.9	64.6	37.0
≥12 years	43.6	7.3*	3.8*	13.8*	10.9*	15.1*	30.2*
missing	0.1	34.3	35.4	19.2	46.2	20.2	32.7
Size of labor unit							
<1500	37.6	1.5*	18.6*	11.7*	17.7*	23.1*	33.6*
1500-2999	28.1	33.0	30.3	38.7	32.5	19.5	27.1
≥3000	34.3	65.4	51.2	49.6	49.8	57.3	39.3
Term gestational days, mean (SD)^a^	282 (9.0)	279* (9.0)	283* (9.3)	277* (9.3)	280* (8.7)	278* (9.7)	278* (9.0)
Gest.diabetes	0.7	3.4*	1.6*	1.5*	1.7*	5.4*	2.1*
Pregest. diabetes	0.5	1.6*	0.7	0.5	1.0*	1.8*	0.8*
Hypertensive disorder	5.2	3.9*	4.2*	1.8*	2.8*	3.7*	5.3
Abruptio placenta	0.5	0.4	0.5	0.5	0.4	0.6	0.5
Placenta previa	0.2	0.2	0.2	0.3	0.3	0.3	0.9*
SGA^b^	4.2	12.4*	8.5*	9.0*	6.9*	10.4*	5.3*
LGA^c^	6.3	2.5*	3.3*	2.3*	3.1*	3.1*	4.4*
Major birth defect	2.4	2.8*	2.7	1.6*	2.7	2.3	2.0
Smoking^d^							
Yes	18.9	0.6*	1.6*	2.0*	2.1*	0.3*	3.4*
No	61.6	62.1	62.9	71.0	69.6	69.2	72.5
Not disclosed	19.5	37.3	35.4	27.0	28.3	30.5	24.1

*Different from non-immigrants (χ^2^ test with P < 0.05).

^a^Term gestational days with standard deviation was calculated in term infants with spontaneous labor onset.

^b^SGA, small-for-gestational age infant, 5th percentile.

^c^LGA, large-for-gestational age infant, 95th percentile.

^d^LGA, large-for-gestational age infant, 95th percentile.

### Overall PTD rates

Over the period under study, 48 191 cases of PTD were noted. Among both immigrants and non-immigrants, 70% of cases of PTD were classified as spontaneous, and 86% were classified as late PTD (weeks 32–36). Overall, the PTD rate was 29% higher among immigrants (6.8%) compared with non-immigrants (5.2%, Table 4). This difference was due to an increased rate of both subtypes of PTD. Filipino women were most likely to experience PTD, with a 52% increased rate compared with non-immigrants, whereas Somali women were the least likely to experience PTD.

**Table 4:** Rates of preterm delivery (PTD) and subtypes among non-immigrants, and immigrants by length of residence

		**PTD**							**Spontaneous PTD**^**a**^							**Non-spontaneous PTD**^**b**^						
**Births**		**Cases**		**By the length of residence (years)**					**Cases**		**By the length of residence (years)**					**Cases**		**By the length of residence (years)**				
				**0-4**	**5-9**	**10-14**	**≥15**	**P**^**c**^			**0-4**	**5-9**	**10-14**	**≥15**	**P**^**c**^			**0-4**	**5-9**	**10-14**	**≥15**	**P**^**c**^
**Population group**	**n**	**n**	**%**	**%**	**%**	**%**	**%**		**n**	**%**	**%**	**%**	**%**	**%**		**n**	**%**	**%**	**%**	**%**	**%**	
Non-immigrants	868 832	45 438	**5.2**						31 492	**3.6**						13 946	**1.6**					
All immigrants	40 709	2753	**6.8**	6.4	6.4	7.3	8.8	<0.001	1935	**4.8**	4.8	4.4	4.6	5.7	0.089	818	**2.0**	1.6	2.0	2.7	3.1	<0.001
Immigrants by country of birth:																						
Iraq	5879	308	**5.2**	5.4	4.6	6.7	3.1	0.691	221	**3.8**	4.0	3.0	4.4	2.1	0.259	87	**1.5**	1.4	1.6	2.3	1.0	0.296
Pakistan	10 096	774	**7.7**	7.0	7.2	7.7	9.3	0.001	493	**4.9**	4.9	4.5	4.5	5.6	0.256	281	**2.8**	2.1	2.7	3.2	3.7	<0.001
Phillippines	5069	404	**8.0**	6.9	8.6	9.7	11.9	<0.001	297	**5.9**	5.3	6.4	7.2	6.6	0.066	107	**2.1**	1.6	2.2	2.5	5.4	<0.001
Somalia	8094	407	**5.0**	5.0	4.4	5.3	12.8	0.026	252	**3.1**	3.4	2.4	2.7	7.7	0.857	155	**1.9**	1.6	2.0	2.7	5.1	<0.001
Sri Lanka	5235	386	**7.4**	6.1	7.9	9.6	10.5	<0.001	273	**5.2**	4.5	5.5	5.8	8.4	0.006	113	**2.2**	1.5	2.4	3.8	2.2	0.003
Vietnam	6336	474	**7.5**	9.3	6.8	5.7	6.8	0.001	399	**6.3**	8.2	6.1	4.3	5.0	<0.001	75	**1.2**	1.0	0.7	1.4	1.7	<0.001

^a^Spontaneous PTD included births <37 completed gestational weeks with spontaneous labor onset or with prelabour rupture of membranes.

^b^Non-spontaneous PTD included births <37 completed gestational weeks with onset by induced labor or by cesarean section.

^c^χ^2^ test for trend by categories of length-of-residence.

### Spontaneous PTD

**Figure 2:**
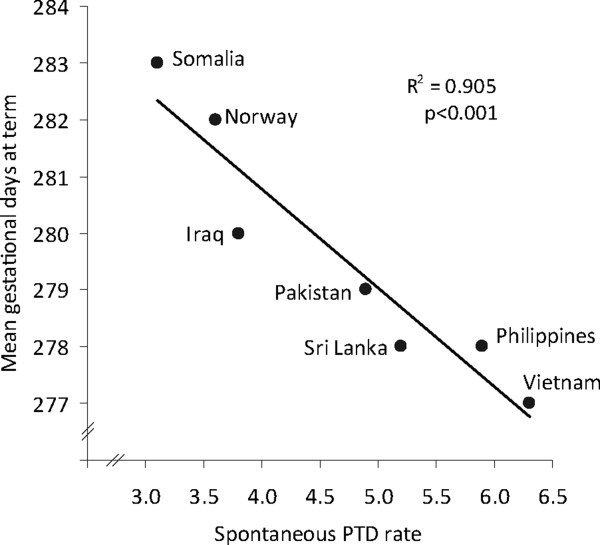
**The relationship between the spontaneous preterm delivery (PTD) rate and gestational days at term according to the country of birth.** Term pregnancies included births at ≥37 completed gestational weeks with spontaneous labor onset.

The observed spontaneous PTD rate among immigrants was 21-57% higher across all categories of residence compared with the rate among non-immigrants (Table 4). Rates were stable across the length-of-residence categories among women from four of six countries; the exceptions were Sri Lankan women (among whom the rate increased) and Vietnamese women (among whom the rate decreased). Spontaneous PTD was most frequent among Vietnamese women (6.3) and the least frequent among Somali women (3.1), whilst the rate was 3.6 among non-immigrants. Although Somali women with the longest residencies (≥15 years) had a high spontaneous PTD rate of 7.7, this trend was non-significant across the length of residence. The spontaneous PTD rate was inversely related to the mean gestational length, in days, in *term* births in each minority group (R^2^ = 0.905, p < 0.001; Figure 2).

**Figure 3:**
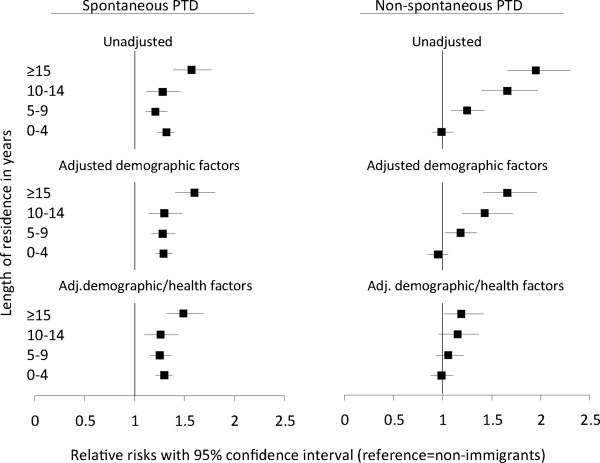
**The estimated relative risks of spontaneous and non-spontaneous preterm delivery (PTD) in non-immigrants (reference line) and in immigrants by length of residence.** The model adjusting for demographic factors included year of delivery, maternal age, parity and size of labor unit. The model adjusting for demographic and health factors included all demographic factors as well as gestational and pregestational diabetes, hypertensive disorder, smoking, small- and large-for-gestational age infant and major birth defects.

In adjusted models, the likelihood of spontaneous PTD among immigrants remained 20-60% higher than among non-immigrants across all categories of residence (Figure 3). This pattern persisted in analyses limited to immigrants only (Table 5). Significant disparities by country of birth remained.

**Table 5:** Adjusted relative risk (RR) of preterm delivery (PTD) and subtypes among immigrants (n = 40 709)

	**PTD**		**Spontaneous PTD**^**a**^		**Non-spontaneous PTD**^**b**^	
	**Adjusted demographic factors**^**c**^	**Adjusted demographic**^**c **^**and health factors**^**d**^	**Adjusted demographic factors**^**c**^	**Adjusted demographic**^**c **^**and health factors**^**d**^	**Adjusted demographic factors**^**c**^	**Adjusted demographic**^**c **^**and health factors**^**d**^
	**aRR [95% CI]**	**aRR [95% CI]**	**aRR [95% CI]**	**aRR [95% CI]**	**aRR [95% CI]**	**aRR [95% CI]**
Length of residence (years)						
0-4 (Reference)	1.00	1.00	1.00	1.00	1.00	1.00
5-9	0.97 [0.88-1.06]	0.93 [0.85-1.02]	0.90 [0.80-1.01]	0.89 [0.80-1.00]	1.15 [0.96-1.38]	1.05 [0.88-1.25]
10-14	1.00 [0.88-1.14]	0.93 [0.83-1.05]	0.86 [0.74-1.00]	0.83 [0.72-0.97]	**1.43** [1.15-1.78]	1.17 [0.94-1.67]
≥15	**1.22** [1.08-1.38]	1.10 [0.98-1.24]	1.07 [0.92-1.24]	1.03 [0.90-1.20]	**1.68** [1.34-2.10]	**1.28** [1.02-1.61]
P trend	0.012	0.391	0.883	0.516	<0.001	0.018
Country of birth						
Iraq (Reference)	1.00	1.00	1.00	1.00	1.00	1.00
Pakistan	**1.29** [1.12-1.48]	**1.26** [1.10-1.45]	1.16 [0.99-1.37]	**1.20** [1.02-1.42]	**1.57** [1.21-2.04]	**1.37** [1.06-1.76]
Phillippines	**1.41** [1.22-1.64]	**1.32** [1.14-1.53]	**1.48** [1.24-1.76]	**1.43** [1.19-1.70]	1.25 [0.94-1.68]	1.08 [0.81-1.44]
Somalia	0.95 [0.82-1.10]	0.89 [0.77-1.03]	**0.82** [0.69-0.98]	**0.82** [0.68-0.98]	1.26 [0.97-1.64]	1.07 [0.83-1.38]
Sri Lanka	**1.31** [1.13-1.52]	**1.29** [1.11-1.50]	**1.32** [1.10-1.58]	**1.34** [1.11-1.60]	1.27 [0.95-1.69]	1.20 [0.90-1.59]
Vietnam	**1.26** [1.09-1.46]	**1.38** [1.19-1.59]	**1.53** [1.28-1.81]	**1.60** [1.35-1.89]	**0.64** [0.47-0.89]	0.81 [0.59-1.12]

^a^Spontaneous PTD included births <37 completed gestational weeks with spontaneous labor onset or with prelabour rupture of membranes.

^b^Non-spontaneous PTD included births <37 completed gestational weeks with onset by inducted labor or by cesarean section.

^c^Demographic factors included the length of residence, country of birth, year of delivery, maternal age at delivery, parity and size of labor unit.

^d^Health factors included gestational and pre-gestational diabetes, hypertensive disorders, placenta disorders, major birth defects, maternal smoking and small- and large-for-gestational age infants.

Bold text indicates significant difference relative to the reference.

### Non-spontaneous PTD

The observed non-spontaneous PTD rate among immigrants increased with the length of residence (p trend <0.001, Table 4). This pattern was found in women from four of the six countries; the exception was for Sri Lankan and Iraqi women; for the latter group a low number of cases destabilized the rate among the longest length-of-residence category. Overall, the observed non-spontaneous PTD rate was highest among Pakistani women (2.8) and lowest among Vietnamese women (1.2), whereas the rate was 1.6 among non-immigrants. The observed non-spontaneous rates by country group were not associated to the average gestational length (data not shown).Also in adjusted models the likelihood of experiencing non-spontaneous PTD increased linearly with longer length of residence (p trend <0.001, Figure 3). After adjusting for demographic factors, recent immigrants (residence of 0–4 years) had similar chances of experiencing non-spontaneous PTD as non-immigrants, whereas immigrants with lengths of residence of 5–9 years, 10–14 years and ≥15 years had 18% (aRR 1.18, 95% CI 1.03,1.35), 43% (aRR 1.43, 95% CI 1.20,1.71) and 66% (aRR 1.66, 95% CI 1.41,1.96) increased chances, respectively, of experiencing non-spontaneous PTD (Figure 3). When we additionally adjusted for health-related covariates, the effect measures were reduced, mainly due to the inclusion of pregestational diabetes and hypertensive disorders. These conditions were positively associated with increasing length of residence in the adjusted model.

In the models that included immigrants alone*,* we observed a similar pattern of results, even after adjusting for the country of birth (Table 5). Relative to immigrants with residence of 0–4 years there was a significant trend with increasing length of residence for non-spontaneous PTD, but not for spontaneous PTD. The association with residence was even stronger for early non-spontaneous PTD (weeks 22–31), which was 33-203% more likely among women with residence ≥5 years. In contrast, the results for late (weeks 32–36) non-spontaneous PTD did not differ from those of the overall analyses.

### PTD rates according to gestational length determination

During the later period of the study, when we had information on gestational length that was determined using both dating methods, PTD rates among immigrants were 7.5% based on the LMP dating and 6.2% based on ultrasound dating (p < 0.001) (data not shown). The difference was due to a 21% reduction in spontaneous PTD rates, whereas non-spontaneous PTD rates were unaffected. Among non-immigrants, the PTD rate did not differ by dating method (both were 5.3%) (data not shown).

## Discussion

### Main findings

In this study, we compared the risk of spontaneous and non-spontaneous PTD among immigrants according to their time in residence with the risk of PTD among the majority population. Our results show that immigrants overall were more likely to experience either PTD subtype than non-immigrants. However, only non-spontaneous PTD was positively associated with length of residence. Among recent immigrants (residence <5 years), the risk of non-spontaneous PTD was similar to the risk among non-immigrants, whereas the risk increased by 18-66% with lengths of residence ≥5 years. Conversely, the risk of spontaneous PTD was not mitigated by length of residence, but varied with country of birth according to the duration of pregnancy in *term* births.

### Increasing risk of non-spontaneous PTD across migrants’ length of residence

The influence of the length of residence on the likelihood of experiencing non-spontaneous PTD was robust across subanalyses and was independent of the age at immigration and the period of arrival. A trend in overall PTD was also present, consistent with findings from Canada [25,29]. However, in our study, no subgroup of immigrants had lower risks of PTD than the host population, thus we did not find any evidence of a ‘healthy migrant’ effect. This could be due to the particular characteristics of immigrants that arrive to Norway. Unlike countries such as Canada, Norway did not allow unskilled labour immigration between 1975 and 1994, at which point Norway became associated to the European Union Single market [30]. Thus, there might have been less pressure towards selection to migration to Norway in comparison to other receiving countries. As to recent immigrants, we did not confirm an elevated PTD risk, as reported from Denmark [11].

We found that the association between the length of residence and non-spontaneous PTD was mainly explained by an increase in maternal and fetal risk factors. Of these, diabetes and hypertensive disorder had the strongest influence. In the general population, studies from the US have shown increasing occurrence of diabetes [22], obesity [21] and cardiovascular risk factors [23] across immigrants’ lengths of residence. A higher risk of illness during pregnancy with longer length of residence has been found in other migrant populations [31]. In pregnancy, diabetes and hypertensive disorders disturb the placental-fetal unit and both are common medical indications for the interruption of pregnancy before term [32,33]. These disorders not only predispose the infant to preterm delivery but also directly contribute to mortality. However, if our findings were simply due to an increase in morbidity, we would expect to observe a similar association with spontaneous PTD, given the many shared determinants of the two subtypes [3,34]. We did not observe this pattern. Our findings could therefore have been influenced also by a more frequent *detection* of risk conditions among women with longer residence. Improvements in the language skills of immigrants, as well as more knowledge on the part of providers, could improve communication and trust between the two. In turn, such processes are likely to affect the reporting and referral of complications [35].

After adjusting for a range of mediating factors, and relative to our reference of Iraqi women, Pakistani women retained an elevated risk of having a preterm obstetric intervention. This finding cannot be readily explained by our data and needs further investigation. Among other country groups, there were no significant differences relative to the reference.

Education level, as a proxy measure for SEP, is closely associated to adverse infant outcome [36]. In our study, information on maternal education level was missing among a high proportion of recent immigrants. Education level is not routinely registered among migrants upon arrival in Norway, but is gathered by questionnaires at a later stage, which explains this finding. The covariate was not included in the final models, as education level among immigrants was not a predictor for PTD in univariable analyses. This does not mean that there is an absence of a socioeconomic gradient in PTD among foreign-born women, but rather that the maternal educational level does not capture it in the same way as among native-born women [33,37].

### Elevated risk of spontaneous PTD among migrants irrespective of the length of residence

Spontaneous PTD receives a great deal of attention because it constitutes the majority of PTD cases [38]. Our results indicate that spontaneous PTD has limited value as an indicator of adverse neonatal outcome in ethnically mixed populations. After adjusting for a range of mediating factors and relative to our reference of Iraqi women, Vietnamese, Philippine, Sri Lankese and Pakistani women retained an elevated risk of having a spontaneous PTD. These minority groups in our sample were more likely to be registered as having experienced spontaneous PTD for two reasons. First, the likelihood of being classified as preterm for most minority groups corresponded with the mean duration of pregnancy in *term* births, suggesting a bias in the use of a uniform cut-off point for PTD. Second, a change in gestational age dating from ultrasound to LMP dating resulted in inflated PTD rates among immigrants, confirming previous findings among non-white women [39]. The change in dating method did not affect non-immigrants, which is consistent with previous findings that a change from LMP to ultrasound dating slightly *increased* rather than decreased the PTD rate [40,41]. As gestational length has a strong genetic basis, this contributed to the persisting high spontaneous PTD rates we found in several minority groups. Somali women retained the lowest risk of all minority groups, which again is likely to be influenced by their longer duration of pregnancy compared to other groups.

### Strengths and limitations of the study

The strengths of this study include the population-based design, with minimal selection bias; the high quality and completeness of the data on exposures (length of residence and country of birth); and the ability to distinguish between the modes of labor onset. The study included births over a 20 year period and although there was a secular increase in preterm caesarean section over this period due to improvements in neonatal care, these changes were adjusted for. Additionally, differential misclassification of the modes of labor onset and the occurrence of preterm prelabor rupture of membranes is believed to be minimal and unlikely to affect the observed associations. However, the present study also had limitations. First, we did not have information on covariates such as maternal height, BMI and previous PTD. In addition, the routine registration of maternal and fetal morbidity can be suboptimal; however, a validation of pregestational diabetes and hypertensive disorders in the MBRN was found to be satisfactory [42,43]. Of SEP indicators, we only had information on maternal education level, and not on other indicators such as income or profession. Second, effects of the length of residence can be difficult to distinguish from cohort effects and the effect of the age at immigration; however, the associations found remained when controlling for these factors. Pakistani and Vietnamese women constituted a high proportion of women with the longest residences; this could have influenced the results for women in this category. Third, the routine determination of gestational length and fetal growth in Norway is interpreted according to majority standards. In addition to affecting preterm rates, it also affected classifications to SGA, and influenced the high prevalence of SGA infants born to foreign-born mothers [11,25]. The alternative; country- or ethnic-specific standards [44], were not available to us, and their implications for clinical care is still controversial [15,16]. To ultimately evaluate the clinical significance of the disparities in preterm subtypes, a determination of the optimal gestational ages of specific groups in terms of infant outcomes would be needed.

## Conclusions

In this study we found that migrants’ risk of non-spontaneous PTD increased with the length of residence in Norway, whereas this was not the case for spontaneous PTD. The increased risk of non-spontaneous PTD among immigrants with increasing length of residence was mainly explained by an increase in registered maternal and infant morbidity. This signifies that post-migration exposures contribute to rather than decrease the burden of preterm birth. This has implications for clinical care regarding the primary prevention of lifestyle and diet-associated pregnancy complications. An improved detection of risk conditions as an expression of improved obstetric care with migrants’ length of residence could also contribute to our findings; however, to clarify this further studies of the indications for preterm obstetric intervention is needed. Ethnic differences in the risk of both PTD subtypes persisted in adjusted models. The efforts to reduce the negative outcomes associated with PTD should focus on preventing the modifiable causes of maternal and fetal morbidity rather than aiming at reducing absolute PTD rates. Similar to other studies, this study stresses the need to distinguish immigrants by length of residence and country of birth to gain insights into ethnic variation in birth outcomes.

## Abbreviations

PTD: Preterm delivery; SGA: Small-for-gestational age infant; LGA: Large-for-gestational age infant.

## Competing interests

The authors declare that they have no competing interests.

## Authors’ contributions

All authors have fulfilled all conditions required for authorship. IKS, AKD and SV designed the study. IKS analysed the data and wrote the first draft of the manuscript. All authors contributed to the interpretation of the results, revised further drafts, and approved the final manuscript.

## Pre-publication history

The pre-publication history for this paper can be accessed here:

http://www.biomedcentral.com/1471-2393/14/239/prepub
